# Telemedicine in Oncology: Delivering on an Overdue Promise in the COVID-19 Era

**DOI:** 10.3389/fonc.2020.578888

**Published:** 2020-09-25

**Authors:** Howard (Jack) West

**Affiliations:** ^1^Department of Medical Oncology, City of Hope Comprehensive Cancer Center, Duarte, CA, United States; ^2^AccessHope, Duarte, CA, United States

**Keywords:** telemedicine, tele-oncology, telehealth, coronavirus, COVID-19

## Abstract

Telemedicine has historically been underutilized in medicine broadly, and specifically in oncology, despite the general availability of the needed infrastructure to offer it as a platform for remote care. The COVID-19 pandemic posed new risks of infection exposure and potentially life-threatening complications, particularly in patients with cancer, created a new setting for cancer care ideally suited for the rapid roll-out of telemedicine for patients with cancer who need regular follow up but in whom live visits may not be critical. In the months since the upheaval of our health care system and wider society in the United States, as well as other countries, our early experience with telemedicine has demonstrated the feasibility of telemedicine for a subset of patients with cancer, facilitated by a removal of regulatory hurdles and payment parity, at least temporarily. At the same time, however, many patients still need to return to the clinic for routine infusions of anti-cancer therapy that obviate much of the value of remote clinic visits, and many patients remain limited by access to hardware, fast and reliable internet, and technical expertise. While we need to address these shortcomings and ensure training of health care professionals in “webside manner” with patients, as well as to work to develop ways to incorporate remote care in the conduct of clinical trials, telemedicine is poised to emerge not merely as an interim solution to a transient challenge but as a valuable tool ideally suited to deliver cancer care efficiently for a subset of patients well suited to adopt this platform.

Prior to our era of coronavirus disease-19 (COVID-19), telemedicine was a clearly underutilized platform, employed by only 2–3% of the population.(1, 2) We have long had the hardware and bandwidth to serve most of the population, while the lack of easy access to specialists in many parts of the United States has represented a clear need (3, 4). Patients are often required to travel long distances for specialty care or because the health care services previously near them have been scaled down or closed entirely. Many oncology clinics are filled with patients reviewing labs or imaging results and then returning home, a process that can consume most or all of a day when considering the drive time, delays in multiple steps, etc. But the barriers of inconsistent reimbursement below parity with live visits, protectionist state medical licensing requirements to obstruct competition with local providers while generating revenue, and the sheer inertia of the *status quo* among powerful incumbents in the dysfunctional United States health care system have hobbled the potential for broad adoption of telemedicine.

The coronavirus pandemic and enforced physical distancing unleashed a perfect storm have made it an ideal setting for telemedicine. With the recognition that merely venturing out from home, particularly to a hospital or clinic with patients and health care professionals (H), is accompanied by real risk of exposure to coronavirus that appears to disproportionately affect patients with cancer and other comorbidities, (5, 6) the latent potential of telemedicine emerged as a solution. To their credit, the Center for Medicare and Medicaid Services (CMS) rapidly developed sweeping changes to federal regulations for telemedicine that guaranteed coverage of 80 services that could be delivered by telemedicine and even telephone-based visits and be reimbursed at parity with that provided for a live visit in the clinic (7, 8). The revised standards also specifically relaxed regulation of HIPAA compliance and states that physicians can communicate with patients through popular and readily accessible platforms like Facebook and Skype. States are currently in the ongoing process of revising and relaxing their restrictions on the practice of telemedicine, while also working to ensure parity of payment for telemedicine with live services in order to both enable patients to receive care from home in order to reduce risk of exposure to coronavirus and to allow HCPs to work from home or other settings apart from those with higher risk of themselves being infected (9).

With this combination of acutely and dramatically demonstrated need along with suddenly reduced or eliminated barriers to adoption, we are in the midst of the catalyzation of telemedicine in cancer care from a promising concept waiting in the wings to a rapidly executed roll-out in private and academic centers alike (10, 11). Translators, additional specialists, and family members even from other parts of the country can potentially be integrated into telemedicine encounters. Importantly, at a time when many centers prohibit family members from accompanying the patient for in person visits in order to reduce risk of virus exposure, telemedicine visits may provide a unique opportunity for family participation. At many centers, scant parking and the requirement for physical distancing of patients in clinic waiting rooms have only exacerbated crowding that may have been present even before coronavirus, making the potential decompression of patients through telemedicine an appealing alternative that reduces spatial concerns.

At City of Hope Comprehensive Cancer Center (COH), we have not only integrated telemedicine as an alternative to live visits for patients in our outpatient cancer clinic, but we have pioneered an array of remote consult services as AccessHope, a wholly owned subsidiary of COH that offers subspecialist expert insight and support for patients as an employer benefit for a growing group of companies. These services include a cancer support line available to patients and staffed by nurses, as well as coordinated live or televideo visits with COH faculty specialists for patients from certain areas of the United States, and larger numbers of patients having case medical records reviewed and a detailed summary of management recommendations offered to local oncologists, with a goal of having patients receive care close to home but with the input of a subspecialist in their tumor type. As oncology becomes increasingly complex and new targets and treatments become available, insight on demand from a subspecialist disease expert can help provide a critical supplement for general oncologists managing patients with ten or more different types of cancer every day. These services may also extend to specialists such as genetic counselors, pathologists, palliative care specialists, and even nutritionists and social workers who may not be readily available in smaller cancer centers.

As valuable as telemedicine has been in the wake of new restrictions borne of concern about coronavirus exposure and the need for physical distancing, there is greater potential as programs for home-based infusions, remote and potentially in-home phlebotomy, and scans at outside facilities become more widely available and utilized.

Clinical research programs have also been hard hit during the COVID-19 pandemic, and telemedicine has the potential to not only help overcome these issues but to confer a sustained greater efficiency to the clinical research enterprise by integrating telemedicine whenever possible, long beyond the immediate pressing concerns around exposure related to person patient visits. Sponsor companies, clinical research organizations (CROs), cancer research cooperative groups, and regulatory bodies are now evaluating the needs to adhere to rigid historical precedents for live visits. A liberalized ability to combine far more judicious live clinic visits with additional telemedicine-based visits for introductory screening, selected follow-up during the trial, and post-treatment monitoring provides an opportunity not only to enable clinical cancer research to continue through the COVID-19 pandemic but also to broaden the availability of trials to a patient population geographically distant enough that more frequent live visits are prohibitive.

Even so, the rapid roll out of telemedicine has also led to a recognition of the shortfalls that limit its use to a subset of patients. First, despite limited programs for home-based infusions of chemotherapy or immunotherapy, the vast majority of patients on regimens of intravenous (IV) therapy must present to their local cancer center or hospital for its administration, making it sensible to pair this with a clinic visit with their oncologist. While some remote digital monitoring tools are increasingly becoming available, physical exams are generally infeasible and limited to a viewed assessment of the patient through a webcam image. Lung auscultation or palpation of a spleen tip remains the province of in-person clinic visits for the foreseeable future.

Other patients have been limited by their lack of sufficient hardware, bandwidth, or technical ability to navigate the sometimes complex multistep processes required to connect with their HCP online. Moreover, the persistence of these challenges may exacerbate disparities in delivery of care as telemedicine services become readily available to only a subset of patients with care. Fewer than two thirds of households headed by a person aged 65 and order have a desktop or laptop, and broadband and other fast internet options are not universal and especially limited among racial minorities (12). But even with access to the hardware and fast internet, many patients – disproportionately those who are older, minorities, and those with limited education – do not have significant experience with navigating online interactions to be considered “digitally literate” (13).

In addition, HCPs working in shared clinic spaces may have difficulty finding an appropriate space to conduct telemedicine visits with sufficient privacy unless exam rooms are converted into makeshift telemedicine workstations. Many physicians and patients alike also have yet to develop skills with artful communication over a webcam and computer screen, especially if technical glitches punctuate some conversations. Beyond these practical barriers, HCPs need time and expertise to develop cultural competencies to work with patients to integrate platform of telemedicine that is foreign to varying degrees for patient and physician alike.

Arguably the leading shortcoming of telemedicine in the setting of oncology is the concern about compromising the personal connection sought between patients with cancer and their medical team, especially their oncologist, over their longitudinal relationship. Some oncologists who recognize the practical advantages of telemedicine nevertheless express hesitation about being unable to maintain a needed rapport with patients during what are often emotionally charged conversations about prognosis, outcome of scans monitoring response to treatment, and goals of care. This is especially true for initial visits with new patients that are especially critical for establishing this relationship, but many oncologists favor live clinic visits even for well established patients, at least in the absence of a true need to keep patients away from others. We should bear in mind, however, that in contrast with our extensive experience with direct patient-physician conversations in the exam room, most of us have experience with telemedicine limited to just months; our “web-side manner” will only improve and diminish the gap between live clinic and telemedicine-based discussions.

What should we expect in the future? One overarching question is whether the momentum of telemedicine is similar to a makeshift offsite COVID-19 facility, a temporary deviation in our practice patterns, or whether telemedicine will become a sustained component of our care. Importantly, it remains to be seen whether the critical reforms that led to recent widespread adoptions, such as reimbursement at parity with live visits, along with relaxation of HIPAA concerns and historically obstructive state licensing requirements, will be rescinded in a world in which an effective treatment or vaccine for coronavirus are readily available. We should hope, however, that as the feasibility and utility of telemedicine has been convincingly demonstrated, these enabling, supportive changes remain to preserve the option of telemedicine for appropriate patients. In that setting, telemedicine will not replace conventional cancer care in the clinic but can coexist as a platform well suited for many patients and cancer physicians. For those who are a significant distance from the clinic, who are clinically stable, and who have the requisite equipment and technical facility, telemedicine will remain a valued method to remain connected with their cancer care team while reducing the time and effort compared to care physically tethered to the clinic. Importantly, the appeal of telemedicine will only increase if programs for in-home infusions and blood collection, as well as remote imaging, become more comprehensive. Our challenges in communication over a screen should also be reduced as patients and HCPs alike gain more experience with remote meetings, which have become the norm in settings from school to the office, as well as in telemedicine.

We should also hope that the increasingly widespread use of telemedicine leads to further refinements. Most telemedicine platforms are not seamlessly integrated with electronic medical record systems like EPIC, but this would be a very helpful development. In addition, to help overcome limits in availability of hardware, bandwidth, and technical ability, patients could potentially receive loaned hardware like a smart phone or tablet, even possibly equipped with limited data service, featuring an icon for immediate and direct access to a telemedicine waiting room, perhaps also pre-loaded with educational videos and even platforms for patients to communicate patient-reported outcomes, new side effects, and changes in disease symptoms. Positive experiences with outpatient clinical care may also open the door to other uses where it remains minimally incorporated, such as in the inpatient setting.

We are currently only capturing an early glimpse of the potential for the still immature practice of telemedicine that has been borne in haste and out of necessity as a response to sudden and dramatic changes in health care due to coronavirus.

## Data Availability Statement

The original contributions presented in the study are included in the article/supplementary material, further inquiries can be directed to the corresponding author.

## Author Contributions

The author confirms being the sole contributor of this work and has approved it for publication.

## Conflict of Interest

HW is employed by AccessHope, a subsidiary of City of Hope.
